# Phylogenetic and phenotypic characterization of *Burkholderia pseudomallei* isolates from Ghana reveals a novel sequence type and common phenotypes

**DOI:** 10.3389/fmicb.2024.1401259

**Published:** 2024-07-09

**Authors:** Kevin L. Schully, Logan J. Voegtly, Gregory K. Rice, Hannah Drumm, Maren C. Fitzpatrick, Francisco Malagon, April Shea, Ming Dong, George Oduro, F. J. Lourens Robberts, Paul K. A. Dartey, Alex Owusu-Ofori, Danielle V. Clark, Regina Z. Cer, Kimberly A. Bishop-Lilly

**Affiliations:** ^1^Austere Environments Consortium for Enhanced Sepsis Outcomes (ACESO), Biological Defense Research Directorate, Naval Medical Research Command-Frederick, Ft. Detrick, MD, United States; ^2^Genomics and Bioinformatics Department, Biological Defense Research Directorate, Naval Medical Research Command-Frederick, Ft. Detrick, MD, United States; ^3^Leidos, Reston, VA, United States; ^4^National Strategic Research Institute, Omaha, NE, United States; ^5^Austere environments Consortium for Enhanced Sepsis Outcomes (ACESO), The Henry M Jackson Foundation for the Advancement of Military Medicine, Bethesda, MD, United States; ^6^Komfo Anokye Teaching Hospital, Kumasi, Ghana; ^7^Independent Consultant, Stellenbosch, South Africa; ^8^CSIR-Crops Research Institute, Kumasi, Ghana; ^9^Department of Clinical Microbiology, Kwame Nkrumah University of Science and Technology, Kumasi, Ghana

**Keywords:** *Burkholderia pseudomallei*, melioidosis, Ghana, Africa, environment, phylogenetic, genome, sequence

## Abstract

Melioidosis is a potentially severe disease caused by the gram-negative soil-dwelling bacterium called *Burkholderia pseudomallei*. The true breadth of the distribution of this tropical pathogen is starting to emerge with environmental and clinical isolates frequently characterized in new countries and regions. Even so, isolates, clinical cases, and genetic data from the continent of Africa remain scant. We previously confirmed the presence of *B. pseudomallei* in the environment of Ghana, unmasking a new area of endemicity for this pathogen. Here, we describe the genetic characteristics of isolates obtained from that environmental survey. Twenty-one isolates were subjected to whole genome sequencing and found to represent three discrete sequence types (ST), one of which was novel, and designated ST2058. Phylogenetic analysis places this novel isolate within a *B. pseudomallei* clade that includes genomes derived from the Americas, although it is closely related to a sub-clade that includes isolates from Africa. Importantly, phenotypic characterization demonstrates common features including API 20NE profiles and *B. pseudomallei* CPS to support existing diagnostics, and susceptibility to standard of care antibiotics often used in the clinical management of melioidosis. These findings add to our knowledge about the presence and distribution of *B. pseudomallei* in Africa and represent the first published genomes out of Ghana.

## Introduction

*Burkholderia pseudomallei* is a gram-negative soil saprophyte and is the causative agent of the disease known as melioidosis. The bacterium was previously described as being sporadically endemic throughout the tropics with areas of endemic concentration in Thailand and Northern Australia responsible for approximately 2,000 deaths per year. However, in Limmathurotsakul et al. (2016) utilized computer modeling to predict a wider distribution and suggested nearly ubiquitous endemicity throughout the tropical latitudes causing significant morbidity and mortality worldwide (Limmathurotsakul et al., 2016). Consistent with that prediction, *B. pseudomallei* has been identified in clinical and environmental specimens in areas not previously known to be endemic, including South Asia (Mukhopadhyay et al., 2018; Jayasinghearachchi et al., 2022), the Caribbean (Hall et al., 2019; Stone et al., 2020), and North America (Salam et al., 2011; Morosini et al., 2013; Torres, 2023).

Even as *B. pseudomallei* was discovered to have a broader geographic distribution around the world, knowledge of the epidemiology and distribution of *B. pseudomallei* in Africa remained relegated to a small number of sporadic cases, mostly exported from Africa, and anecdotal accounts (Wall et al., 1985; Cuadros et al., 2011; Sarovich et al., 2016; Steinmetz et al., 2018; Mabayoje et al., 2022). Efforts to remedy that knowledge gap have recently increased through campaigns to raise awareness, increase diagnostic capabilities, and identify *B. pseudomallei* in the environment (Steinmetz et al., 2018; Birnie et al., 2019, 2022; Savelkoel et al., 2023). We recently conducted an environmental survey of five rice paddies in South-central Ghana and confirmed the presence of *B. pseudomallei* in Ghana through standard culture and biochemical approaches, and found that these isolates were susceptible to standard antimicrobial therapies for melioidosis (Oduro et al., 2022). Although the epidemiological picture of *B. pseudomallei* in Africa is starting to emerge, genetic data remains scant with only 69 of the 6,817 isolates in PubMLST associated with Africa (assessed on December 26, 2023). Of these, only nine genome assemblies are available; notably two (id: 4412 and 4413) originated from Burkina Faso, the northern neighbor of Ghana. The other seven assemblies originated from Nigeria. GenBank has 144 complete *B. pseudomallei* (taxonomy ID: 28450) genomes available but none are from Africa (Supplementary Table 1).

**FIGURE 1:**
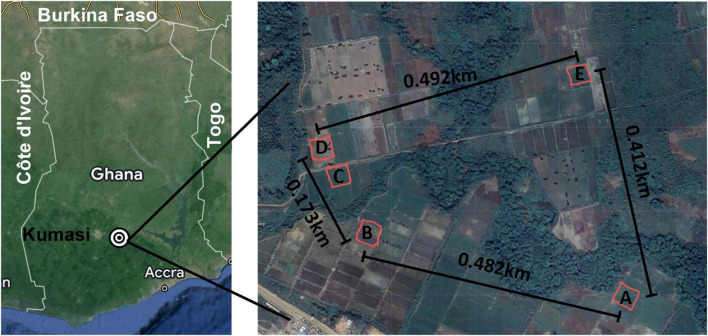
Sampling area. **Left:** Map of Ghana with major reference points included and the sampling area indicated with concentric circles. **Right:** Each of the five sites is indicated at scale with the distance between each noted on the map. A mobile phone field measurement tool, GPS Fields Area Measure (FARMIS; Lithuania), was used with an Apple iPhone X (Apple Inc.; Cupertino, CA, USA) to record the Global Positioning Coordinates (GPS Coordinates., 2023) of each sampling site by tracing the coordinates while walking around the demarcated perimeters of each sampling site. The resulting field shape coordinates were exported as KML files. The distance between each site was determined using the Distance Calculator.

*B. pseudomallei* has a large, dynamic, and genetically diverse genome owing to horizontal gene transfer and site-specific recombination occurring at integration hotspots located throughout its genome (Tuanyok et al., 2008). This genomic plasticity makes genetic characterization a complicated but essential component of the description of potentially novel strains. Multi-locus sequence typing (MLST) is a commonly used method for characterizing the epidemiology of *B. pseudomallei*, the diversity within environmental samples, and origin of clinical specimens (Godoy et al., 2003; Dale et al., 2011; Roe et al., 2022). However, the granularity and resolution that whole genome sequencing (WGS) provides for genomic characterization remains the most accurate method, particularly for molecular epidemiological studies related to outbreak surveillance and for investigations of biogeography and diversity. In our previous study, we identified Ghana as an endemic area for *B. pseudomallei* and described the phenotypic characteristics of isolates obtained from one of five sites (Figure 1, site E) of this environmental survey (Oduro et al., 2022). In this follow-up study, we describe the genetic characteristics of isolates obtained from 5 sampling sites (Figure 1) from which 21 isolates were found to represent 3 discrete sequence types (ST), 1 of which was novel. These findings add to our knowledge about the presence and distribution of *B. pseudomallei* in Africa and represent the first published genomes that originated in Ghana.

## Materials and methods

### Environmental sample collection

**FIGURE 2:**
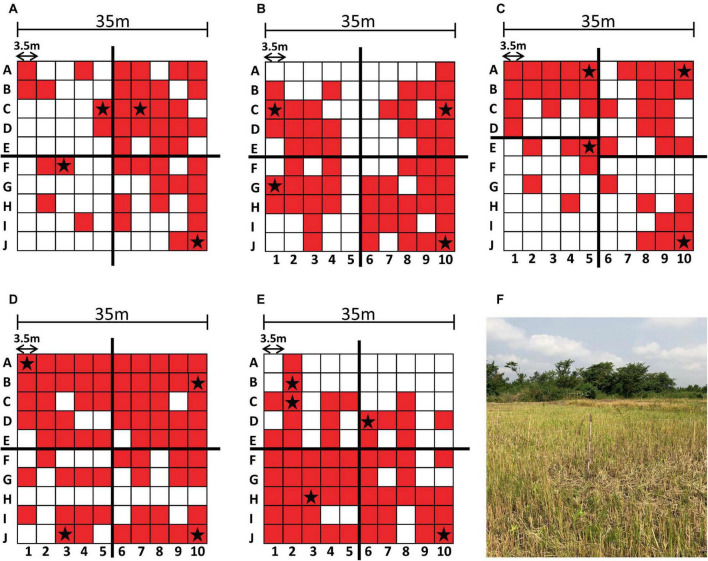
Sampling results and environment. Each sampling grid is depicted with the PCR-positive sampling points shaded in red and the sequenced genomes indicated with a star **(A–E)**. The augmented quadrants are shown in **(C)**. Sampling site **(A)** is shown as a representative example of the sampling environment of each sampling site **(F)**.

Soil samples were collected in January 2021 from 100 points within each of five sites, each 35 m^2^, around the Ashanti region of Ghana as previously described and shown in Figure 1 (Oduro et al., 2022). Sampling environment and sampling site details are presented in Figure 2F and Supplementary Table 2, respectively. Soils were enriched for *B. pseudomallei* using a combination of methods previously described. The enriched samples were preserved by combining 1.2 ml of the resulting cultures with 0.3 ml of 80% sterile glycerol and frozen at −80°C for shipment and storage. Frozen cultures were thawed and used directly to prepare crude DNA extracts for screening by PCR. PCR-positive samples were colony purified on Ashdown’s agar and pure cultures of suspected *B. pseudomallei* were utilized for DNA isolation and phenotypic characterization.

### PCR and culturing

Briefly, crude DNA extracts were prepared by lysing 100 μl of frozen stocks at 95°C for ten minutes. Cell debris was pelleted by centrifugation and 2 μl of the resulting supernatant was used as template in the PCR reaction as previously described (Novak et al., 2006). Each extract was analyzed in duplicate, and each reaction plate contained positive controls including *B. pseudomallei* strain Bp82 crude extract and purified Bp82 genomic DNA, as well as negative controls including *B. thailandensis* strain E264 crude extract and water. To identify extracts containing *B. pseudomallei* genomic DNA, cutoff values were generated by plotting the results of true positive samples versus true negative samples using Graphpad Prism (version 9.0, Boston, Massachusetts USA).^1^ Frozen stocks from sites determined to be positive by PCR were used to inoculate Ashdown’s agar plates which were incubated at 35°C for 96 h. Colonies presenting a morphology consistent with *B. pseudomallei* (i.e., flat, wrinkled, purple colonies) were selected for genetic characterization.

### Genomic DNA isolation

Suspected *B. pseudomallei* colonies were inoculated into 5 ml tryptic soy broth (TSB) and incubated at 37°C overnight while shaking. The following day, 1 ml was withdrawn, the cells were pelleted, and genomic DNA was isolated using the MasterPure Complete DNA and RNA Purification Kit (LGC Biosearch Technologies; Middleton, WI) according to the manufacturer’s instructions. Purified genomic DNA was quantified using Qubit dsDNA BR assays (ThermoFisher Scientific; Waltham, MA, USA) and a Qubit 4 fluorometer. DNA integrity was evaluated by electrophoresis using Genomic DNA ScreenTape, genomic reagents and a TapeStation 4150 (Agilent Technologies; Santa Clara, CA, USA).

### Sample selection for sequencing

Each sampling site of 35 m^2^ was divided into a 10 × 10 grid of 3.5 m^2^ (Oduro et al., 2022). To select colonies that represented each site, we divided each site into four quadrants of 25 sampling points. Due to the uneven distribution of PCR-positive sites in sampling site C (Figure 2), the upper left quadrant was reduced to 20 points and the lower left quadrant was expanded to 30 points (Figure 2). The highest quality DNA sample from each quadrant was selected for sequencing. If a given quadrant had only one sample, then that sample was chosen regardless of DNA concentration. Specific locations are indicated with a star in the grids of Figure 2.

### Sequencing

Short-reads shotgun libraries were prepared using NEBNext Ultra II FS DNA Library Prep Kit for Illumina (New England Biolabs; Ipswich, MA, USA) following the manufacturer’s instructions. Briefly, 26 μl of DNA at 2–4 ng/μl was first fragmented enzymatically via 20 min incubation at 37°C. Next, hairpin sequencing adaptors, containing 5′-dT overhangs and a U ribonucleotide in the hairpin loop, were added to the fragmented DNA by ligation, and subsequently cleaved at the U sites. The libraries were then amplified and indexed by PCR using NEBNext Unique Dual Indexes. Prior to sequencing, the libraries were evaluated for quality using Agilent D1000 kit (Agilent Technologies; Santa Clara, CA, USA). The libraries that passed quality control were then quantified using Qubit dsDNA BR assay (ThermoFisher Scientific; Waltham, MA), pooled, and sequenced using a NovaSeq 6000 S4 Reagent Kit, v1.5 300 cycles, and a NovaSeq 6000 sequencer (Illumina; San Diego, CA, USA). Long-reads libraries were prepared using Ligation kits with native barcodes (Oxford Nanopore Technologies, Oxford, UK) following the manufacturer’s instructions. Briefly, 8 μl of DNA at ∼50 ng/μl was first polished for ligation using NEBNext FFPE DNA Repair Mix. The end-prepped DNA was then indexed by incubation with ONT Native Barcodes and Blunt/TA Ligase Master Mix (NEB). The indexed libraries were then pooled and ligated to ONT AMXII sequencing adaptors using NEBNext Quick T4 DNA ligase. Free adapters and short library fragments were eliminated by cleaning with AMPure beads (Beckman Coulter; Brea, CA) and ONT Long Fragment Buffer. Finally, the libraries were sequenced using a MinION_Flow Cell (R9.4.1) and a MinION-MK1C sequencer (Oxford Nanopore Technologies, Oxford, UK).

### Quality control and *de novo* assembly of sequencing data

The NovaSeq data were processed using MetaDetector as previously described (Adhikari et al., 2024). Briefly, via this pipeline, reads were trimmed and filtered using BBDuk (v38.96) (Bushnell, 2014) and the resulting data were assembled using SPAdes (v.3.15.3) (Bankevich et al., 2012). The reads were mapped back to the contigs using BBMap (v38.96) (Bushnell, 2014), and the reads and contigs were classified using DIAMOND BLAST against NCBI’s non-redundant protein database (nr accessed February 10, 2023) (Buchfink et al., 2015). The ONT reads were assembled using dragonflye (v1.0.14) (Kolmogorov et al., 2019). In addition to these two methods, trimmed and filtered NovaSeq reads were further subsampled to 20 million (M) reads using seqtk (v1.3-r106) and combined with ONT reads for a hybrid assembly using UniCycler (v0.5.0) (Wick et al., 2017).

### Genome closure

The most cohesive UniCycler assemblies were selected from each site (except for SiteE where Drangonflye assembly was used) and manually extracted using Bandage (v0.9.0) (Wick et al., 2015). The extracted genomes were validated using Qiagen CLC Genomics Workbench (v23.0.2)^2^ by mapping the subsampled 20M NovaSeq reads and Oxford Nanopore Technologies (ONT) reads against the draft genome and using the *Analyze Contigs* function to identify problematic regions and *Basic Variant Detection* to identify regions with differences in the reads and contigs (CLC Microbial Genomics Module 23.0). The problematic regions and differences were then manually resolved. This process was repeated until there were no problematic regions or differences identified, resulting in a high-quality draft genome.

### Genome annotation and multilocus sequencing typing (MLST)

Genome annotation was performed using EDGE Bioinformatics (v2.4.0 BDRD 2023FEB09) (Li et al., 2017) with Prokka (v1.14.0) (Seemann, 2014). Virulence factors were identified using EDGE with ShortBRED (v0.9.4M) (Kaminski et al., 2015), antibiotic resistance genes were identified using EDGE with Resistance Gene Identifier (RGI v5.0.1; database v3.0.7) (Jia et al., 2017), and insertion sequences were identified using BLAST against the ISFinder database (accessed 2023-05-04) (Siguier et al., 2006). The MLST database for *B. pseudomallei* was downloaded via CLC on March 28, 2023, and May 04, 2023. CLC Type with MLST Scheme function was used with the SPAdes contigs, and the high-quality draft genomes, to identify sequence types for each of the samples. CLC Genomics Workbench was used to pull the core genome multilocus sequence typing (cgMLST) database and perform the cgMLST to determine a possible cgST. The cgMLST database has 4,071 alleles and requires a minimum fraction of 0.50 to have confidence in typing.

### Comparative genomic analyses

A total of 1,777 complete assemblies were downloaded from GenBank and nine assemblies associated with Africa were downloaded from PubMLST on May 04, 2023. Snippy (v4.6.0) (Seemann, 2015) was used to generate an alignment of the core single nucleotide polymorphism (SNP) genomes using *B. pseudomallei* Mahidol-1106a (Assembly Accession GCA_000756125.1) from Thailand as the reference. Using the core SNP alignment, IQ-Tree (v1.6.10) (Nguyen et al., 2015) was used to generate a Maximum Likelihood tree with automatic model testing using TVM+F+ASC+G4 and 1,000 bootstrap and BEAST (v1.10.4) was used to generate a Bayesian tree using HKY model and 1M MCMC chains. Mauve (v2.4.0) (Darling et al., 2004) was used to perform a whole genome alignment of the three high-quality draft genomes and reference *B. pseudomallei* Mahidol-1106a.

### Phenotypic characterization of *Burkholderia pseudomallei* isolates

Phenotypic characterization such as enzymatic activity and carbohydrate utilization were evaluated using the Analytical Profile Index system, specifically API 20NE (BioMerieux; Cambridge, MA, USA). Antibiotic sensitivity testing (AST) using the disk diffusion method and detection of *B. pseudomallei* capsular polysaccharide by the Active Melioidosis Detect (InBios International Inc, Seatle, WA, USA) lateral flow immunoassay were conducted as previously described (Oduro et al., 2022).

## Results

### Screening of environmental samples for *B. pseudomallei*

Soil cultures were generated from a soil sampling expedition in Ghana using the selective enrichment process described by Trinh and colleagues (Trinh et al., 2019). We first sought to screen these cultures to down-select from our 500 soil samples, using PCR-specific for *B. pseudomallei*. Real time PCR targeting *orf2* of the *B. pseudomallei* Type III Secretion System is considered 100% specific for *B. pseudomallei* because this region is not present in any near neighbor species (Novak et al., 2006). Ct values of true positive results derived from crude extracts of *B. pseudomallei* strain Bp82, prepared as described in the “Materials and methods” section, were plotted against the negative values to generate a Receiver Operator Characteristic (ROC) curve to identify a cutoff value. As depicted in Supplementary Figure 1, the area under the curve (AUC) was 1.0 [95% Confidence Interval (CI) 1.0 to 1.0]. The ROC curve identified a PCR cutoff value for 100% sensitivity and 100% specificity (Table 1). By applying a cutoff value of >30.47 to DNA extracts from each of the 100 points across each site, we found the positive rates as follows: Site A, 45%; Site B, 56%; Site C, 43%; Site D, 70% and Site E, 60%. These results confirm our previous observation that *B. pseudomallei* is ubiquitous throughout the Ashanti region of Ghana, although the distribution is not even (Figure 2 and Supplementary Table 2) (Oduro et al., 2022).

**TABLE 1:** Potential cutoff values indicating PCR-confirmed *B. pseudomallei*.

Ct Cutoff	Sensitivity %	95% CI	Specificity %	95% CI
> 24.55	100.0	77.19 to 100.0%	91.67	64.61 to 99.57%
> **30.47**	**100.0**	**77.19 to 100.0%**	**100.0**	**75.75 to 100.0%**
> 37.28	92.31	66.69 to 99.61%	100.0	75.75 to 100.0%

The sensitivity and specificity are provided for three potential cutoff Ct values, as well as the confidence interval (CI) for each of those values. The Ct value for 100% sensitivity and 100% specificity is presented in bold.

### Sequencing and *de novo* assembly results

We performed whole-genome sequencing using both short read and long read platforms on 21 isolates from the five sampling sites around the Ashanti Region of Ghana (Figures 1, 2) with *de novo* assembly of the resulting sequencing data (Supplementary Table 3). Each genome from the same site was found to be very similar (i.e., a minimum of 97.92% nucleotide identity percentage); therefore, one representative genome from each site was deemed to be sufficient rather than producing high-quality drafts of duplicate genomes. To that end, five genomes from four sampling sites including one each from SiteA_10J, SiteC_5E, SiteD_1A, SiteD_10J, and SiteE_6D were manually closed to high quality draft genome status (Supplementary Table 4). An isolate from Site B was not chosen due to a high degree of sequence similarity to isolates from Site A.

### MLST sequence typing and phylogenetic analyses

Isolates from three of the sampling sites represented previously identified MLST Sequence Types (ST). ST930 was found in site A and site E and is represented by isolates found in four countries including the soil of Nigeria, located to the East of Ghana (Savelkoel et al., 2023). ST1749 from sampling site C is represented by a single isolate from Mexico.^3^ The genome of isolates from sampling site D was found to have a novel sequence type, now designated ST2058 (Table 2).

**TABLE 2:** Detailed information on MLST sequence types of the *B. pseudomallei* isolates.

Sample point	Strain name	Chromosome 1 length (bp)	Chromosome 2 length (bp)	lipA	gltB	lepA	gmhD	narK	ace	ndh	MLST sequence type	Core genome sequence type	cgMLST kmer fraction
SiteA-3F	GHA3F			5	1	2	3	1	1	1	ST930		
SiteA-5C	GHA5C			5	1	2	3	1	1	1	ST930		
SiteA-7C	GHA7C			5	1	2	3	1	1	1	ST930		
SiteA-10J	GHA10J	4,034,701	3,217,548	5	1	2	3	1	1	1	ST930	cgST1	0.48
SiteB-1C	GHB1C			5	1	2	3	1	1	1	ST930		
SiteB-1G	GHB1G			5	1	2	3	1	1	1	ST930		
SiteB-10C	GHB10C			5	1	2	3	1	1	1	ST930		
SiteB-10J	GHB10J			5	1	2	3	1	1	1	ST930		
SiteC-5A	GHC5A			6	1	2	2	1	1	1	ST1749		
SiteC-5E	GHC5E	4,061,312	3,203,862	6	1	2	2	1	1	1	ST1749	cgST812	0.52
SiteC-10A	GHC10A			6	1	2	2	1	1	1	ST1749		
SiteC-10J	GHC10J			6	1	2	2	1	1	1	ST1749		
SiteD-1A	GHD1A	4,051,091	3,134,872	6	1	2	13	2	1	3	ST2058	cgST571	0.46
SiteD-3J	GHD3J			6	1	2	13	2	1	3	ST2058		
SiteD-10B	GHD10B			6	1	2	13	2	1	3	ST2058		
SiteD-10J	GHD10J	4,051,098	3,134,866	6	1	2	13	2	1	3	ST2058	cgST571	0.46
SiteE-2B	GHE2B			5	1	2	3	1	1	1	ST930		
SiteE-2C	GHE2C			5	1	2	3	1	1	1	ST930		
SiteE-3H	GHE3H			5	1	2	3	1	1	1	ST930		
SiteE-6D	GHE6D	4,046,104	3,215,367	5	1	2	3	1	1	1	ST930	cgST1	0.47
SiteE-10J	GHE10J			5	1	2	3	1	1	1	ST930		

**FIGURE 3:**
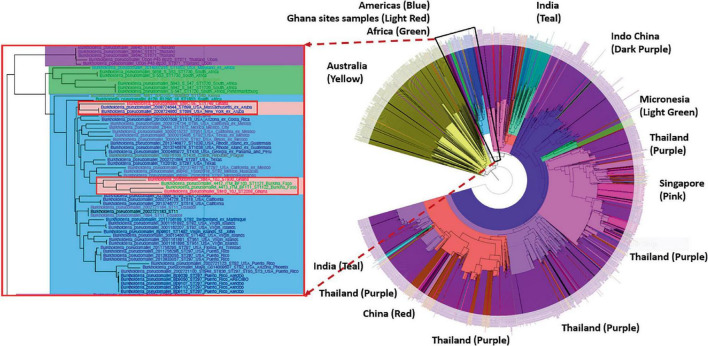
Phylogenetic analyses of the *Burkholderia pseudomallei* genomes from five different sites. **Left:** The genomes from Ghana grouped within a clade associated with isolates from the Americas. **Right:** The isolates from Site A (ST930) and the new ST2058 from Site D grouped within a sub-clade that includes isolates from Ghana-neighboring Burkina Faso. ST1749 from Site C grouped closely with isolates from Aruba.

Phylogenetic analyses showed that the genomes from Ghana grouped within a clade associated with isolates from the Americas. The isolates from site A (ST930) and the two isolates designated with the new strain type ST2058 grouped within a sub-clade that includes isolates from Ghana-neighboring Burkina Faso, whereas the isolate with strain type ST1749 from Site C grouped closely with isolates from Aruba (Figure 3). We also observed that all genomes obtained from these sites contained the *Yersinia*-like fimbrial (YLF) (also referred to as fimbria/pilus outer membrane usher protein) gene. The presence of YLF is in agreement with a 2014 study by Gee et al. (2017) that established that YLF gene is associated mainly with isolates from locations other than Australia.

### Phenotypic characterization

We conducted phenotypic characterization, including enzymatic activity, carbon utilization, capsular polysaccharide (CPS) detection and AST analyses of representative isolates of each of the STs including Site C, point 5E (C5E) representing ST1749, Site E point 2C (E2C) representing ST930 found in sites A and E. We selected two isolates representing our new ST from sampling site D (D1A and D10J). These isolates were from the farthest points on the sampling grid (Figure 2) and were selected due to minor differences in their genome sequences that could have translated into phenotypic variations. Each isolate was oxidase positive, was CPS positive, and produced an API 20NE profile of 1156577, one of the most common *B. pseudomallei* profiles (Table 3) (Amornchai et al., 2007). Each isolate was also susceptible to common antibiotics used in the management of melioidosis (Table 4).

**TABLE 3:** Phenotypic characterization and results.

	*B. pseudomallei* API profile 1156577																					
	**NO3**	**TRP**	**GLU**	**ADH**	**URE**	**ESC**	**GEL**	**PNPG**	**GLU**	**ARA**	**MNE**	**MAN**	**NAG**	**MAL**	**GNT**	**CAP**	**ADI**	**MLT**	**CIT**	**PAC**	**OX**	**CPS**
GHC5E	+	–	–	+	–	–	+	–	+	–	+	+	+	–	+	+	+	+	+	+	+	+
GHD1A	+	–	–	+	–	–	+	–	+	–	+	+	+	–	+	+	+	+	+	+	+	+
GHD10J	+	–	–	+	–	–	+	–	+	–	+	+	+	–	+	+	+	+	+	+	+	+
GHE2C	+	–	–	+	–	–	+	–	+	–	+	+	+	–	+	+	+	+	+	+	+	+

Bacterial identification by API 20NE. For each strain, the API profiles are shown alongside their reaction to *B. pseudomallei* capsular polysaccharide (CPS) LFI. NO_3_, potassium nitrate reduction; TRP, L-tryptophane (indole production); GLU, D-glucose fermentation; ADH, L-arginine DiHydrolase; URE, urea (urease production); ESC, esculin hydrolysis; GEL, gelatin hydrolysis; PNPG, 4-nitrophenyl-βD-galactopyranoside (β-galactosidase production); GLU, D-glucose assimilation; ARA, L-arabinose assimilation; MNE, D-mannose assimilation; MAN, D-mannitol assimilation; NAG, N-acetyl-glucosamine assimilation; MAL, D-maltose assimilation; GNT, potassium gluconate assimilation; CAP, capric acid assimilation; ADI, adipic acid assimilation; MLT, malic acid assimilation; CIT, trisodium citrate assimilation; PAC, phenylacetic acid assimilation; OX, cytochrome oxidase. + indicates a positive test.

**TABLE 4:** Antibiotic susceptibility profiles.

Organism	Strain	Zone of inhibition (mm) for antimicrobial agent					
		**CAZ (30 μ g)**	**IPM (10 μ g)**	**MEM (10 μ g)**	**DO (30 μ g)**	**AMC (20/10 μ g)**	**SXT (1.25/23.75 μ g)**
*B. pseudomallei*	GHC5E	29	28	25	27	27	31
	GHD1A	29	37	26	29	27	31
	GHD10J	28	35	26	26	28	31
	GHE2C	30	35	27	29	28	31

Antibiotic susceptibility profiles for isolates from Ghana. Each disk and its content (in parentheses) are provided, as are the zones of inhibition for amoxicillin-clavulanic acid (AMC), ceftazidime (CAZ), doxycycline (DO), imipenem (IPM), meropenem (MEM), and trimethoprim-sulfamethoxazole (SXT).

## Discussion

Here, we present the first genotypic and second phenotypic analyses of *B. pseudomallei* isolates obtained from the soil of Ghana. Out of 21 genomes sequenced, we identified three discrete sequence types including a unique ST, hereby designated ST2058. SNP-based phylogenetic analyses of the core genomes show that isolates from Ghana cluster within the American Clade, and an African sub-clade, of known *B. pseudomallei* genomes. These results are consistent with other studies and a recent hypothesis proposing an African origin of *B. pseudomallei* in the Americas, potentially seeded by the Atlantic slave trade (Chewapreecha et al., 2017). Additionally, the YLF gene cluster was universally present in all of the sequences from this study. The YLF cluster is typically found in isolates from Southeast Asia, as opposed to the *Burkholderia thailandensis*-like flagellum and chemotaxis biosynthesis (BTFC) gene cluster, which are often found in the genomes of isolates from Australia (Tuanyok et al., 2008). These genomes can now be added to the 20 *B. pseudomallei* genomes from the Americas confirmed to have YLF by BLAST searches (Supplementary Table 5) in future *B. pseudomallei* phylogeographic studies (Gee et al., 2017). Because YLF is overrepresented in clinical isolates, it is presumed to be a virulence factor, although its significance in disease is unknown due to a lack of correlation between YLF and disease severity (Sarovich et al., 2014). Animal studies to determine the virulence of these and other African isolates would be a worthwhile endeavor in follow-up to this work.

The two-step selective enrichment process described by Trinh and colleagues (Trinh et al., 2019) proved to be a robust and reproducible method to culture *B. pseudomallei* from these complex environmental samples. This method employed the traditional consensus guidelines but also capitalized on *B. pseudomallei’s* unique ability to utilize erythritol as its sole carbon source. While near neighbor species were more-than-likely present in the original soil sample, every colony we examined that morphologically resembled *B. pseudomallei* on Ashdown’s agar was positively confirmed to be *B. pseudomallei* by PCR. We recommend this selective enrichment method be adapted more widely for future environmental surveys.

The development of sustainable rice farming is expanding in Ghana. In the Ashanti Region, the southern part of Ghana, more than one hundred hectares of land have been manually developed for rice crops since 2004. Considerable development has occurred to date and the Inland Valley Rice Development Project (IVRDP) aims to expand land development by another 1,500 ha, including Ejisu-Juaben, Ahafo Ano South, Ahafo Ano North, and Ejura-Sekyedumasi districts of the Ashanti region. These developments will lead to land use changes including clearing of sites, leveling, and terracing of rice fields, development of water control structures and development of access tracks (Ministry of Food and Agriculture, 2019). The IVRDP Rice development project has led to significant positive effects on communities, including income generation and education. Changes in land use may also have contributed to changes seen in human disease prevalence such as malaria, a disease that is readily recognized by routine laboratory testing (Mpianing, 2016). However, undifferentiated clinical diseases not subjected to specific laboratory testing may remain uncharacterized, as evidenced by a recent report out of a hospital in the region that details 200 gram-negative clinical isolates obtained over a six-month period that does not include *B. pseudomallei* (Agyepong et al., 2018).

The emergence of new pathogens or unmasking of previously underappreciated pathogens provides the opportunity to increase the capacity to diagnose and treat the infections they cause (Birnie et al., 2022). We performed phenotypic analyses that demonstrate that these novel isolates conform to common characteristics such as CPS production detectible by the Active Melioidosis Detect diagnostic, and one of the most common *B. pseudomallei* metabolic profiles differentiated by API 20NE (Table 3) (Amornchai et al., 2007; Houghton et al., 2014). Each isolate was also susceptible to common antibiotics used in the management of melioidosis (Table 4).

In conclusion, environmental testing plays an important role in defining geographical regions with increased risk of melioidosis, as well as to provide unique genomes for in-depth phylogenetic analyses and epidemiological investigations. Through soil sampling and genomic analyses, we identified a novel strain type of *B. pseudomallei* in Africa that contains a putative virulence factor typically associated with clinical isolates in Thailand. Environmental samples in this region represent a rich, but as-yet-untapped source for future endemicity studies.

## Data availability statement

The datasets presented in this study can be found in online repositories. The names of the repository/repositories and accession number(s) can be found here: https://www.ncbi.nlm.nih.gov/, PRJNA1078842.

## Author contributions

KS: Conceptualization, Formal analysis, Funding acquisition, Investigation, Methodology, Project administration, Resources, Supervision, Visualization, Writing – original draft, Writing – review & editing. LV: Data curation, Formal analysis, Methodology, Writing – review & editing. GR: Data curation, Formal analysis, Investigation, Software, Writing – review & editing. HD: Conceptualization, Data curation, Formal analysis, Investigation, Methodology, Project administration, Resources, Software, Supervision, Validation, Writing – review & editing. MF: Data curation, Formal analysis, Investigation, Software, Writing – review & editing. FM: Data curation, Formal analysis, Investigation, Methodology, Resources, Software, Supervision, Writing – review & editing. AS: Data curation, Formal analysis, Investigation, Writing – review & editing. MD: Data curation, Formal analysis, Investigation, Writing – review & editing. GO: Conceptualization, Investigation, Methodology, Project administration, Resources, Supervision, Writing – review & editing. FR: Conceptualization, Formal analysis, Investigation, Methodology, Resources, Supervision, Writing – original draft, Writing – review & editing. PD: Investigation, Methodology, Project administration, Resources, Supervision, Writing – review & editing. AO-O: Data curation, Formal analysis, Investigation, Supervision, Writing – review & editing. DC: Investigation, Resources, Supervision, Writing – review & editing. RC: Conceptualization, Data curation, Formal analysis, Investigation, Methodology, Project administration, Resources, Software, Supervision, Validation, Visualization, Writing – original draft, Writing – review & editing. KB-L: Conceptualization, Data curation, Formal analysis, Investigation, Methodology, Project administration, Resources, Software, Supervision, Validation, Visualization, Writing – original draft, Writing – review & editing.

## Footnotes

1
www.graphpad.com

2
https://digitalinsights.qiagen.com/

3
https://pubmlst.org

## Author disclaimer

KB-L, RC, and KS were federal employees of the United States government. This work was prepared as part of their official duties. Title 17 U.S.C. 105 provides that “copyright protection under this title is not available for any work of the United States Government.” Title 17 U.S.C. 101 defines a US Government work as work prepared by a military service member or employee of the US Government as part of that person’s official duties. The views expressed in this work reflect the results of research conducted by the authors and do not necessarily reflect the official policy or position of the Department of the Navy, Department of Defense, nor the United States Government.
