# Supplementary material for: Phylogenetic and phenotypic characterization of Burkholderia pseudomallei isolates from Ghana reveals a novel sequence type and common phenotypes
Source: Front Microbiol. 2024 Jul 9;15:1401259. doi: 10.3389/fmicb.2024.1401259 (PMC11264198; doi:10.3389/fmicb.2024.1401259)
Supplement: Supplementary file 1 [file Data_Sheet_1.pdf]

Supplemental Materials:

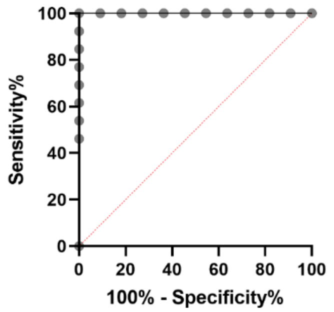

Figure S1. Receiver Operator Characteristics (ROC) curve for PCR screen. *Ct values of true positive samples versus true negative samples were plotted to generate a ROC curve. The area under the curve (AUC) and confidence intervals are represented.*

Observations	Area	P value	95% Confidence Interval	
24	1.0	<0.0001	1.0	1.0

Table S1. Distribution of *B. pseudomallei* complete genomes published per country.

Country	Count
Australia	30
China	3
Czech Republic	1
Ecuador	2
Hong Kong	1
India	11
Japan	2
Malaysia	8
Micronesia	7
Pakistan	1
Papua New Guinea	4
Singapore	8
Sri Lanka	9
Switzerland	1
Taiwan	3
Thailand	12
USA	20
Venezuela	1
Viet Nam	1
Unknown	5
Not applicable	5
No geo location	9
Total:	144

Table S2. Details of each sampling site.

Sampling Site	Soil pH	Soil Temp °C (at 30 cm)	Air Temp °C (Start/Finish)	Sampling Duration (mins)	% Positive
A	4.60±0.42	32.0±2.16	30/32	72	45
B	5.12±0.86	29.75±1.71	26/28	72	56
C	5.02±0.61	28.6±0.50	29/32	85	43
D	4.96±0.49	31.75±1.26	30/30	124	70
E	4.5±0.49	29.75±5.42	31/32	66	60

At each sampling site the soil temperature and pH weres measured at 4 sampling points within the grid (the four corners) using a 3-in-1 Soil Moisture Light and pH Acidity Tester (Longruner, Shenzen, China). Both were collected at a depth of 30 cm and are presented as the average of four sampling points (the four corners). The ambient temperature was recorded at the start and end of the sampling process using the Weather.com mobile phone application (The Weather Company; Brookhaven, GA) with an Apple iPhone X (Apple Inc.; Cupertino, CA).

Table S3. General sequencing statistics of Illumina and ONT sequencing data

	Illumina NovaSeq short reads		ONT long reads		
Sample	Number of raw reads	Number of reads post QC	Number of raw reads	Total number of bases	N50 of reads
SiteA-3F	950,657,912	52,796,578	32,458	178,218,722	8,886
SiteA-5C	641,180,254	36,337,696	25,289	194,902,678	12,927
SiteA-7C	1,096,404,214	68,341,964	28,966	140,342,966	7,915
SiteA-10J	445,763,362	40,049,058	9,294	59,581,893	12,784
SiteB-1C	600,505,584	47,692,014	14,404	109,811,449	13,603
SiteB-1G	317,330,636	40,796,466	4,615	21,897,279	8,502
SiteB-10C	257,223,720	23,980,830	8,877	38,886,980	7,497
SiteB-10J	727,167,368	59,699,152	17,967	90,312,596	8,217
SiteC-5A	220,388,886	14,747,970	3,304	25,732,783	14,454
SiteC-5E	263,938,700	14,284,640	3,838	34,717,237	17,228
SiteC-10A	177,474,560	11,012,808	2,573	11,876,882	10,070
SiteC-10J	192,551,984	12,079,260	1,882	10,962,742	10,192
SiteD-1A	216,828,592	15,056,902	2,543	16,855,217	13,471
SiteD-3J	261,765,210	15,353,822	3,453	20,679,972	11,864
SiteD-10B	53,488,414	4,935,586	1,876	12,337,695	11,533
SiteD-10J	175,033,338	10,912,358	4,290	43,862,782	19,957
SiteE-2B	474,158,648	35,191,154	10,795	75,914,359	12,635
SiteE-2C	575,030,902	40,637,002	16,246	112,328,769	12,900
SiteE-3H	293,319,470	24,834,392	9,708	43,721,617	7,703
SiteE-6D	379,275,752	46,730,120	23,576	158,068,447	12,529
SiteE-10J	118,464,364	19,184,776	2,534	7,671,397	7,213

Table S4. Statistics of *de novo* assemblies

	SPAdes (Illumina NovaSeq short-reads alone)				DragonFlye (ONT long-reads alone)				Unicycler (Hybrid: short-reads and long-reads)			
**Sample**	**Number of contigs**	**N50**	**Longest Contig**	**Total Length (nt)**	**Numer of contigs**	**N50**	**Longest Contig**	**Total Length (nt)**	**Number of contigs**	**N50**	**Longest Contig**	**Total Length (nt)**
**SiteA-3F**	318	42,224	145,985	7,145,532	2	4,034,291	4,034,291	7,240,221	-	-	-	-
**SiteA-5C**	338	37,584	146,036	7,129,554	4	4,035,567	4,035,567	7,207,233	-	-	-	-
**SiteA-7C**	274	52,336	174,007	7,139,208	3	4,035,402	4,035,402	7,242,804	-	-	-	-
**SiteA-10J**	178	86,885	246,865	7,123,716	26	481,815	1,203,635	7,214,798	38	966,491	1,129,402	7,261,790
**SiteB-1C**	238	59,060	187,413	7,135,660	3	2,251,712	3,218,057	7,271,835	25	953,575	1,700,052	7,243,680
**SiteB-1G**	165	90,230	246,865	7,122,420	-	-	-	-	119	613,941	1,103,915	7,204,119
**SiteB-10C**	173	86,329	246,865	7,121,308	24	27,858	56,899	505,876	90	637,920	1,763,465	7,206,454
**SiteB-10J**	193	82,626	246,865	7,131,526	17	1,278,572	1,662,739	7,241,766	24	1,137,856	1,620,115	7,226,974
**SiteC-5A**	245	73,772	207,700	7,192,811	-	-	-	-	235	419,320	1,069,562	7,290,298
**SiteC-5E**	228	75,322	207,699	7,181,150	3	59,138	38,638	130,260	12	1,900,327	3,203,855	7,315,349
**SiteC-10A**	227	79,863	495,142	7,181,392	-	-	-	-	257	381,096	1,105,663	7,242,896
**SiteC-10J**	233	78,117	207,749	7,191,699	-	-	-	-	329	256,801	597,388	7,244,228
**SiteD-1A**	166	93,728	295,375	7,074,245	-	-	-	-	111	524,397	1,423,635	7,156,976
**SiteD-3J**	275	46,599	140,581	7,089,927	-	-	-	-	293	150,291	383,102	7,182,316
**SiteD-10B**	166	88,734	289,932	7,072,080	-	-	-	-	229	314,137	712,708	7,124,016
**SiteD-10J**	167	97,525	295,375	7,077,104	44	163,475	306,201	4,872,130	30	869,671	3,133,337	7,184,007

**SiteE-2B**	178	82,602	292,172	7,122,233	10	1,062,808	2,068,853	7,245,609	6	2,561,350	3,177,849	7,251,890
**SiteE-2C**	307	46,122	134,115	7,140,861	7	4,002,554	4,002,554	7,242,815	93	660,926	833,121	7,294,880
**SiteE-3H**	172	86,883	246,865	7,119,720	97	38,251	102,621	2,860,528	142	399,315	795,329	7,071,807
**SiteE-6D**	248	56,057	173,994	7,162,343	2	4,049,946	4,049,946	7,268,009	-	-	-	-
**SiteE-10J**	163	88,608	329,333	7,124,120	-	-	-	-	246	266,559	885,484	7,157,384

Table S5. Twenty *B. pseudomallei* genomes from the Americas

Assembly Accession	Strain	Geo location
GCF_001318245.1	Bp1651	USA
GCF_030297255.1	GTC3P0254T	USA
GCF_000959345.1	PB08298010	USA: Arizona
GCF_002111145.1	2010007509	USA: Arizona ex Costa Rica
GCF_002111205.1	2013746777	USA: California
GCF_002111105.1	2002734728	USA: California
GCF_002111365.1	2008724758	USA: California
GCF_002110945.1	2008724734	USA: California ex Mexico
GCF_002111045.1	3000015237	USA: California ex Mexico
GCF_002110965.1	2011756295	USA: Florida ex Trinidad
GCF_002111305.1	3000465972	USA: Georgia ex Panama and Peru
GCF_002111285.1	3000047530	USA: Illinois ex Mexico
GCF_002111125.1	2008724860	USA: New York ex Aruba
GCF_002111225.1	2013746811	USA: Ohio
GCF_002111025.1	2013833055	USA: Puerto Rico
GCF_002111265.1	2013833057	USA: Puerto Rico
GCF_002111325.1	2002721123	USA: Puerto Rico
GCF_002111005.1	2013746878	USA: Rhode Island ex Guatemala
GCF_002111245.1	2013746877	USA: Rhode Island ex Guatemala
GCF_002111385.1	3000015486	USA: Texas ex Mexico
